# Impact of COVID-19 on Immunization Services for Maternal and Infant Vaccines: Results of a Survey Conducted by Imprint—The Immunising Pregnant Women and Infants Network

**DOI:** 10.3390/vaccines8030556

**Published:** 2020-09-22

**Authors:** Anja Saso, Helen Skirrow, Beate Kampmann

**Affiliations:** 1 The Vaccine Centre, Faculty of Infectious and Tropical Diseases, London School of Hygiene & Tropical Medicine, London WC1 7HT, UK; anja.saso@lshtm.ac.uk; 2 Vaccines and Immunity Theme, MRC Unit the Gambia at LSHTM, Banjul P.O. Box 273, Gambia; 3 Department of Primary Care and Public Health, School of Public Health, Imperial College London, London W6 8RP, UK; h.skirrow@imperial.ac.uk

**Keywords:** COVID-19, pandemic, vaccines, maternal immunization, global health, questionnaire, survey, qualitative, neonatal, infant, IMPRINT

## Abstract

The COVID-19 pandemic response has caused disruption to healthcare services globally, including to routine immunizations. To understand immunization service interruptions specifically for maternal, neonatal and infant vaccines, we captured the local experiences of members of the Immunising Pregnant Women and Infants Network (IMPRINT) by conducting an online survey over 2-weeks in April 2020. IMPRINT is a global network of clinicians and scientists working in maternal and neonatal vaccinology. The survey included discrete questions to quantify the extent of disruption as well as free-text options to explore the reasons behind reported disruptions. Of the 48 responses received, the majority (75%) were from low-and-middle-income countries (LMICs). Of all respondents, 50% or more reported issues with vaccine delivery within their country. Thematic analysis identified three key themes behind immunization disruption: “access” issues, e.g., logistical barriers, “provider” issues, e.g., staff shortages and user “concern” about attending immunization appointments due to COVID-19 fear. Access and provider issues were more commonly reported by LMIC respondents. Overall, respondents reported uncertainty among parents and healthcare providers regarding routine immunization. We conclude that further quantification of routine vaccination disruption is needed, alongside health service prioritization, logistical support and targeted communication strategies to reinforce routine immunizations during the COVID-19 response.

## 1. Introduction

By mid-April 2020, the Coronavirus disease-2019 (COVID-19) had spread across all continents with two million cases and 120,000 deaths reported worldwide [1]. Risk factors for adverse outcomes had already been established [2,3] with healthy women of childbearing age, newborn babies and infants generally considered to be less severely affected by the severe acute respiratory syndrome coronavirus-2 (SARS-CoV-2) than other age groups [4,5]. However, as the pandemic has progressed, the associated disruption of public services has caused significant collateral damage with particular implications for maternal and child health [6,7].

There are now growing concerns over the severe impact of COVID-19 on the provision of vital immunization services [8,9], although the consequences are yet to be systematically evaluated. Finding the balance between guarding against the spread of COVID-19 versus controlling well known preventable diseases has proven to be delicate and difficult. For instance, recent modelling has predicted that not maintaining routine childhood immunization in Africa will lead to more deaths than possible COVID-19 deaths associated with visits to vaccination clinics [10]. The World Health Organisation (WHO), in conjunction with UNICEF, GAVI and the Sabin Vaccine Institute, has undertaken two online immunization “pulse” surveys. The first of over 800 national and subnational immunization experts, completed in April 2020, focused on understanding the disruption caused by COVID-19 to routine delivery of the expanded programme on immunization and mass vaccination campaigns [11,12]. The second, published in June 2020, after our own survey was completed, also aimed to understand the extent of disruption, although it had fewer respondents [13]. Based on their first survey, the WHO warned that COVID-19 was beginning to disrupt life-saving immunization services around the world, putting millions of children—in rich and poor countries alike—at risk of diseases such as diphtheria, measles and polio [11]. By the end of May, mass vaccination campaigns against measles were beginning to be cancelled in some countries for fear of contracting COVID-19 when visiting health services [14,15]. The ability to deliver routine vaccines will also have been affected by redeployment of healthcare workers to tackle COVID-19.

To gather a swift snapshot of the global picture and to explore the changes in immunization services experienced by local frontline staff, we initiated an online survey of the members of the IMmunising PRegnant women and Infants NeTwork (IMPRINT) in mid-April 2020. IMPRINT is a UK-funded global network of stakeholders working within basic science, immunology, vaccinology, social sciences, industry, public health and national and international policy, who are investigating the biological and implementation challenges to the use of vaccines in pregnancy and the early postnatal period [16]. Given the special interests of IMPRINT, our survey had a specific focus on immunization services for pregnant women and newborns, but also included questions on routine infant and toddler vaccination schedules. With its 290 members based in 51 countries worldwide, this network provided a unique forum to collate personal insights into the “collateral damage” caused by COVID-19, specifically related to the delivery of local and national immunizations, allowing us to further compare the situation in high income (HICs) versus low and middle income countries (LMICs).

## 2. Materials and Methods

### 2.1. Study Design and Participants

A cross-sectional survey was conducted using an online questionnaire accessible to all members of the international Immunising Pregnant Women and Infants Network (IMPRINT) via the IMPRINT website. The network currently has 290 members in 51 countries, including 32 (63%) LMICs and 19 (37%) HICs, similar proportionally to the distribution worldwide [17]. The questionnaire was distributed via an electronic link to all email addresses stored in the encrypted, secure IMPRINT membership database. A brief explanation of the purpose of the study and assurance of the confidentiality of the data was outlined in the body of the email. The survey was available for completion from 15 April 2020 to 30 April 2020, with a second reminder email sent four days before the survey closed. There were no specific eligibility criteria but only one submission per participant was permitted.

### 2.2. Survey Description

The study questionnaire had several short sections covering: (1) the professional background of the respondent, (2) country-specific data on COVID-19 epidemiology, (3) national policies for routine delivery of vaccines to pregnant women, newborns and infants, (4) experience and perception of issues relating to vaccine delivery for these target groups due to the COVID-19 pandemic and (5) availability of local, national and/or international immunization guidelines during this period. Each section of the questionnaire included a set of items in which the respondents were asked to choose a predefined answer listed after a question or statement. In addition, Section 4 and Section 5 included questions inviting participants to provide a further free-text response. Progression to the next set of questions was not possible before answers to all the preceding questions had been registered.

The questionnaire is appended in the Supplementary Materials.

### 2.3. Survey Validation

Given that this survey was created for the purpose of this project and addresses rare (albeit serious) issues in an unprecedented context, we were not able to utilise previously validated questions. Nevertheless, the survey was developed and trialled by a core cohort of IMPRINT global health experts (healthcare professionals and/or research scientists). Input from professionals in other unrelated specialties, including social scientists, helped to inform user feasibility. Difficulties with wording or comprehension were resolved within the core group and, after a consensus was reached, alterations were subsequently made to establish the final version of the survey. This pilot phase, therefore, streamlined the questions as well as indicated that the average response time was 10–15 min.

### 2.4. Ethics Approval and Data Management

This was an IMPRINT members-only survey, circulated via the members’ website and no formal ethical approval was sought. The UK National Health Service (NHS) Health Research Authority decision tool was used and determined that the study did not require review by an NHS Research Ethics Committee. Members could decide freely whether to contribute or not and, by participating, they implied their consent. Experienced researchers facilitated the surveys and ethical considerations for survey research were followed; as for other social science research methods, this involved informing potential participants of the purpose of the research and how their contribution would be used, as well as assuring confidentiality and appropriate use of sensitive material. The study was compliant with general data protection regulation and followed published recommendations on conducting good quality survey research [18,19]. Email addresses were given by survey respondents, although any identifiable data was anonymised for the data analysis (undertaken in Microsoft Excel, Microsoft Corporation, Redmond, WA, USA). Members of the IMPRINT network automatically consent to sharing contact details, such as email addresses, with the other members when joining the network; this is a core element of the network enabling fruitful communication and collaboration. Details can be found on the IMPRINT website, https://www.imprint-network.co.uk [16].

### 2.5. Survey Analysis: Quantitative

The percentage of respondents was calculated across different occupations and countries of origin. The percentage of survey participants who reported issues in the delivery of maternal, neonatal and infant vaccines was calculated and stratified according to HICs versus LMICs, classified according to Development Assistance Committee (DAC) criteria [17].

Quantitative analysis initially involved cleaning the survey data to check for discrepancies between answers from respondents who were from the same country, relating to the type of vaccine delivered and the site of vaccination administration. If discrepancies were found, they were resolved by first checking what answers healthcare providers had given. If these were consistent, and also validated by the published country schedules [20], then they were included in the analysis. Answers were recoded if they were different from healthcare providers’ answers in the same country and did not correlate with the published country schedules. No qualitative free-text responses were amended.

#### Survey Analysis: Qualitative

Participant free-text responses on issues around vaccination during the COVID-19 pandemic were analysed by the three authors following thematic analysis principles [21], through a phenomenological lens. This strategy has been used effectively in previous qualitative or mixed-methods studies that have similarly analysed free-text comments from surveys [22,23]. A traditional content analytical [24] approach (including word and phrase repetitions) was applied to identify common issues to maternal, neonatal and infant/toddler vaccination delivery and key patterns across settings. Having discussed these issues and come to a group consensus, the authors thereby developed a framework of three overarching themes from the free-text responses. The data was subsequently reanalysed to identify further associated subthemes. Based on these themes (both primary and subthemes), the free-text answers were re-reviewed a final time and categorised according to the immunization type (maternal, neonatal and infant/toddler) or country of origin (HICs versus LMICs). Different versions of the framework were shared between the authors multiple times, ensuring that the final framework was representative of all respondents’ answers within the three identified key themes. Example quotes from respondents were assigned to the different subthemes to substantiate the findings.

Participant free-text responses on regional, national and international guidelines were first analysed to identify any specific referenced guidelines or official recommendations, and subsequently to establish any potential differences between responses from HIC and LMIC participants.

The analysis was conducted in a precise, consistent and exhaustive manner, to guide appropriate interpretation and identification of key thematic issues. It adhered to Standards for Reporting Qualitative Research (SRQR) guidelines [25].

## 3. Results

### 3.1. Quantitative Findings

#### 3.1.1. Participants

**Figure 1:**
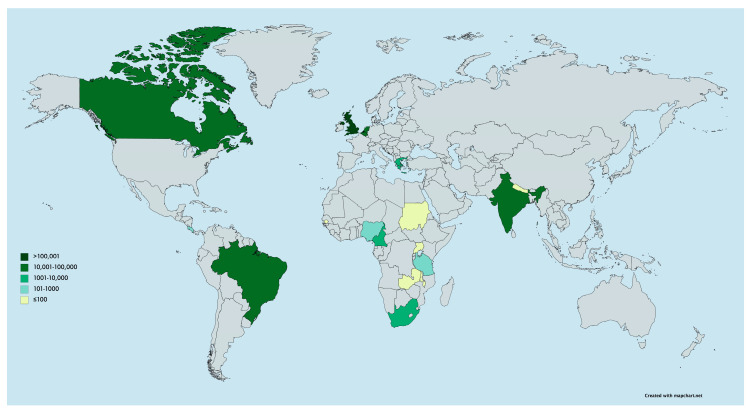
Map of the worldwide distribution of survey respondents and total number of COVID-19 cases at the time of survey completion (created with mapchart.net).

Out of a total 290 IMPRINT members who potentially had access to this survey, 48 responses were received within the limited 2-week period (response rate of 17%), representing 18 different countries. Most respondents were healthcare professionals and based in either public or private settings (Table 1). They were reporting from a range of countries, with the majority from LMICs (75%); answers were received from 36 members based in 13 LMICs and 12 members based in 5 HICs. Please refer to Supplementary Materials, Table S1, for a breakdown of respondents’ occupations by country-of-origin. The official numbers of COVID-19 cases at the time of the survey in each country varied across settings (Figure 1).

#### 3.1.2. Routine Vaccine Schedules and Delivery Locations Prior to COVID-19

To assess different pathways of care involved in the administration of immunizations to pregnant women, newborns and infants or toddlers, we asked questions relating to the recommended vaccine schedule for each group.

*Maternal vaccines:* All participants reported that tetanus toxoid vaccines are routinely given to pregnant women in their country of origin, either as a single vaccine or combined with diphtheria and/or pertussis antigens (note, the Netherlands have only recently rolled out their maternal TdaP programmes) [26]. Despite being recommended by the WHO for antenatal use worldwide, influenza and pertussis vaccines were mainly reported to be part of antenatal schedules in HICs, with the exception of South Africa, India and Costa Rica (although predominantly facilitated in the private sector). Maternal immunizations were reported to be primarily administered by midwives, nurses or doctors in antenatal clinics, across all settings.

*Neonatal and infant/toddler vaccines:* Birth dose vaccines were reported to be part of the routinely delivered national schedules, mainly in LMICs, and were consistent with published schedules [20]. In LMICs, both neonate and infant vaccines were reported to be primarily delivered through an expanded programme on immunization (EPI) clinics, whilst infant and toddler vaccines in HICs were provided mainly through family doctors or paediatricians.

#### 3.1.3. Changes to Vaccine Delivery since the COVID-19 Pandemic

Of all respondents, 50% or more reported disruption in either maternal or infant/toddler vaccine delivery (Table 2); this was a problem identified similarly across both LMICs and HICs. Issues with the delivery of vaccines to newborns were described by 33% (*n* = 16) of all survey respondents, though this was higher among those based in LMICs (42%, *n* = 15). Among those respondents who did not describe any issues, a proportion chose the answer “Don’t know” for whether they knew if immunization services were disrupted, including 9, 8 and 6 respondents in the context of maternal, newborn and infant/childhood vaccine delivery, respectively.

### 3.2. Qualitative Findings

#### 3.2.1. Issues around Vaccine Delivery and Uptake

Overall, survey respondents conveyed a feeling of uncertainty due to the pandemic, independent of the setting, which extended into many services in their countries, including those related to routine immunizations. Three key themes, and several associated subthemes, emerged across all locations and immunization types: “access” issues due to the lockdown and social isolation, as well as logistical difficulties; “provider” issues, including changes to clinics, staff shortages, lack of personal protective equipment (PPE) and vaccine supply problems; and “user” concerns, primarily fear of acquiring COVID-19 and broader vaccine hesitance.

**Figure 2:**
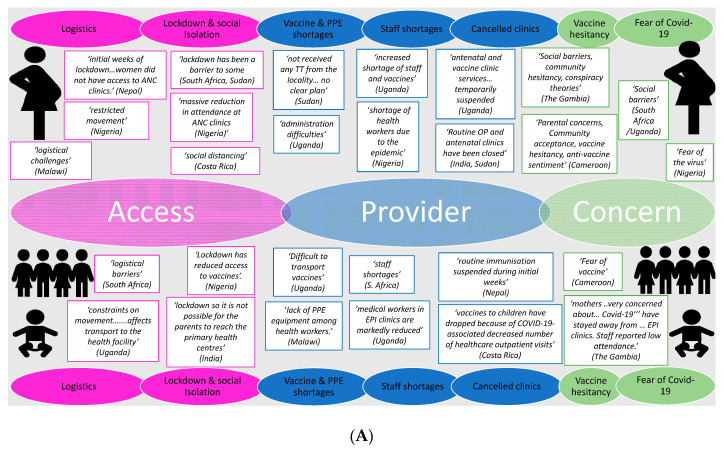

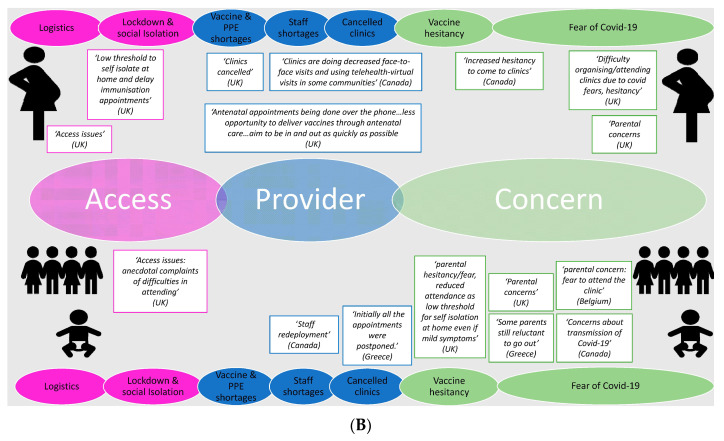
Factors affecting maternal (top of panel), neonatal and infant (bottom of panel) vaccine delivery as reported by respondents from (**A**) low-and-middle-income countries (LMICs) and (**B**) high-income countries (HICs). Note: Selected quotes are included from participant responses to substantiate the chosen themes and subthemes. ANC, antenatal care; TT, tetanus toxoid; PPE, personal protective equipment; COVID-19, coronavirus disease 2019.

Among the “access” issues noted, “lockdown and social isolation” was the most frequently reported barrier. Lockdown measures and social distancing were described as reducing access for pregnant women and families/infants, precluding them from easily reaching antenatal clinics and primary health care centres (to complete the expanded programme on immunization schedule), respectively. “Provider issues”, specifically disruptions to clinics, were reported by most participants, although staff shortages were described more frequently in the context of newborn, infant and toddler vaccines, compared to vaccines for pregnant women (Figure 2).

There were also some differences between respondents from LMICs compared to HICs: the barriers described in LMICs were more skewed towards provider issues, with cancelled clinics and unavailability of vaccines observed irrespective of vaccine types or target groups. By contrast, several respondents from HICs reported changes in the clinic format, for example telephone or virtual consultations being set up, rather than definitive cancellation or suspension of services. Moreover, there were minimal reports of logistical problems and PPE/vaccine shortage among HIC participants. Instead, “concern” issues were reported more frequently as a barrier in this setting, predominantly “fear of COVID-19”, particularly with regards to neonatal, infant and toddler vaccine uptake; barriers to maternal vaccine delivery, however, were associated with all three key issues. Finally, although vaccine hesitancy and social concerns were reported by some respondents, regardless of vaccine type, the phrases “conspiracy theory” and “anti-vaccine (or anti-vaxx) sentiment” were mainly associated with some LMICs (Figure 2).

#### 3.2.2. Regional, National and International Recommendations and Guidelines

Participants from LMICs who responded to these questions primarily referenced WHO international guidelines [27], with some answers describing how their governments had adapted these recommendations accordingly to their setting. Sub-Saharan African participants, for example, specifically pointed out “the application of social distancing and hygiene at immunization sessions as well as scheduling of sessions with few persons to prevent overcrowding” (Nigeria) and encouragement for the “widespread use of facial mask and hand sanitizers in public places” (Cameroon). Similarly, a Nepalese participant described a statement by their Ministry of Health encouraging routine immunization services to continue: “To Maintain Social Distancing, To identify any contraindications to vaccination, PPE provision to Vaccinator”. Only one LMIC respondent (India), however, cited a specific national or regional guideline [28].

Participants from HICs also referenced the WHO guidelines (in the European context) [29] as well as national guidelines or official documents issued by their government, including in the UK [30,31] and Canada [32,33]. The Canadian guideline was released early in the course of the pandemic and specifically described prioritization of vaccines, with the highest importance placed on routine immunizations in infants under 6 months of age. Respondents from the UK also cited guidelines or statements released by professional bodies, including the Royal College of Obstetricians and Gynaecologists for antenatal and postnatal services (although specific vaccines are not mentioned) [34], Royal College of Paediatric and Child Health [35], Royal College of Nurses [36] and Royal College of General Practitioners [37], all of whom issued advice urging continuation of national immunization campaigns.

Other measures or recommendations described were aimed at the local or community level. Participants from LMICs gave more anecdotal evidence, for example, “sensitization and awareness creation of caregivers also to report suspect cases for COVID-19 in their communities to allow for detailed investigation and prevention of community spread” and “recommendations around use of SMS reporting to monitor and track session implementation across the health facilities” (both by Nigerian respondents). Similarly, participants from HICs described innovative measures and community outreach solutions to overcome vaccine delivery barriers, particularly limited access and disruptions to service provision; this included virtual or teleclinics, drive-through vaccine schemes and novel locations for vaccine administration (“maternity outpatients being set up at Tottenham Hotspur football ground so women don not need to go to hospital” in London, UK).

Participants from HICs also referenced more official communications to parents or families, quoting advice issued by general practitioners or community clinics: “we are trying to reassure families that appointments are being staggered and social distancing is being practiced in the waiting areas and increased cleaning practices are in practice” and “parents are advised to stick to the program unless they cannot come to the clinic because someone has a cold or other signs of acute respiratory infection”. Links were provided by some respondents to information sent to parents highlighting the consequences of delayed vaccination [38]. No similar examples of parental communication were provided by LMIC respondents.

## 4. Discussion

The IMPRINT survey has provided a snapshot and “big picture” impression of the impact of COVID-19 on maternal and infant immunization services at the grassroots level, within a global context. At the time of our survey, the epidemic was in full swing in HICs and precautionary measures were being taken across LMICs. Our findings support the concerns of healthcare professionals and organisations worldwide about the significant indirect or “collateral” implications of the global COVID-19 pandemic on the provision of services for pregnant women and children. This is predicted to have enormous consequences to maternal and child health outcomes in both the short- and long-term [7].

Whilst there is public concern about immunization services in times of COVID-19, accurate country-specific data remain poorly available, most notably from LMICs [8,11]. Our stakeholder survey included both HICs and LMICs and found that more than half of respondents reported disruption in routine maternal and childhood vaccination programmes. This is consistent with the World Health Organisation’s (WHO) first online immunization survey of over 800 immunization experts, including representatives of Health Ministries and global health organizations from over 100 countries, which reported that over 50% of the expanded programme on immunization programmes had been disrupted [11,12]. The repeat WHO survey conducted in June 2020 found that immunization programme disruption remained an issue for the majority of respondents [13]. Similarly, in recent correspondence from Pakistan, one of the only published reports providing this level of detailed data in a LMIC, the average number of daily immunization visits (accounting for all antigens) are reported to have decreased by more than half during the lockdown compared with baseline, with the steepest decline at the start of the pandemic in Pakistan in April 2020 [39]. There is also growing evidence from England, Scotland and the United States that the COVID-19 pandemic response has caused vaccination rates to decline even within HICs [40,41,42].

Our survey explores issues as perceived not only by experts but also healthcare providers and researchers, at the grass roots level of vaccine delivery. This grassroots perspective complements the WHO immunization surveys whose respondents worked at national or subnational levels [13]. Moreover, it conveys the concerns of stakeholders from LMICs, specifically Sub-Saharan African, representing 75% and 65% of the total participants, respectively. This has enabled us to explore potential differences between HICs and LMICs. Although several studies have emerged modelling the trajectory of the outbreak in African countries [43], the voice of health care providers and governing bodies in these settings has been less audible and there is a distinct lack of large-scale data or more granular evidence from national immunization programmes. This may be because, to date, the epicentre of the pandemic has primarily been within Asia, Europe and the Americas, with lower case numbers within Sub-Saharan African countries, with the exception of South Africa. As the epidemiology shows, however, the pandemic is still evolving and cases in sub-Saharan Africa are increasing daily [1]. Finally, our survey is unique in reporting issues not only around delivery of the infant expanded programme on immunization vaccines but also extending to immunization services for pregnant women, providing insights into the provision of antenatal care where these vaccines might be administered. Pregnant women represent a vulnerable group, often overlooked during epidemics, as recently seen during the Ebola outbreak [44].

Whilst the overarching sentiment expressed by the respondents was uncertainty, we identified three key vaccine delivery issues relating to (i) access, (ii) provider and (iii) user concerns, each with different associated subthemes. These are largely consistent with the main reasons attributed by experts, to date, to the disruption of global immunization services, primarily highlighting a combination of vaccine demand and supply factors [45]. More specifically, the WHO survey outlined the contribution of: (1) parental reluctance to leave home due to restrictions on movement, a lack of information or fear of COVID-19, (2) poor availability of health workers due to travel restrictions, lack of PPE and redeployment to pandemic response duties and (3) transport delays and problems getting vaccine supplies to clinics [11]. These themes are entirely in keeping with our results.

Although similar issues were broadly reported by all respondents, irrespective of immunization and location, there appeared to be some differences between HICs and LMICs, implying that tailored strategies are needed. “Parental concern” was more frequently reported from HICs respondents, particularly fear of contracting COVID-19 by attending healthcare settings. In comparison, respondents from LMICs more commonly reported “access” and “provider” issues; indeed, difficulties secondary to “vaccine shortages” and “logistics” were primarily described within LMICs and were only rarely mentioned by HIC respondents. Our finding is supported by reports from UNICEF, stating that the pandemic response to COVID-19 has caused logistical delays to vaccine shipments worldwide, namely in LMICs [45].

The consequences of interruptions to routine immunization programmes has the potential to be widespread and catastrophic [7,11]. At least 80 million children under the age of one are estimated to be at risk from vaccine preventable diseases due to missing out of routine immunizations [11] with measles a particular concern [15]. Previous infectious disease outbreaks (including Ebola) were also associated with increased cases of vaccine-preventable diseases such as measles [46]. In the recent outbreak in the Democratic Republic of Congo, three times as many people died from measles than from Ebola [47]. Moreover, lockdown has exposed and exacerbated existing immunization inequities and uptake barriers, both within and between different countries [9]. A recent detailed benefit–risk analysis, modelling the impact of missed childhood vaccinations in Africa, found that the benefit of averting vaccine-preventable infections far outweighed any possible COVID-19 morbidity and mortality associated with immunization clinic visits [10].

Our study has some clear limitations. Although our geographical reach was very wide, the number of respondents per location is small and represents only a convenience sample. In particular there were a small number of respondents (*n* = 12) from HICs. More detail on disruption issues was given from the United Kingdom (UK) respondents, which is then reflected with more comments in Figure 2B being from the UK. A further limitation is that the overall response rate from the IMPRINT network was quite low (17%), although this is likely due to the limited time period given to respond, in order to primarily capture the initial concerns and attitudes towards vaccine delivery, as a “snapshot” at the start of the pandemic. Further surveys would benefit from involving more grass-roots participants based in different locations, including a comparison of rural versus urban settings. Nevertheless, our survey represented 18 nationalities worldwide, across 5 continents, and included a range of stakeholders, based in clinical, public health or research departments. Non-response bias could also have occurred in this study; important responses by participants from particular demographic groups, occupations or countries, or those too busy to respond, could be missing and therefore their views are not accounted for within the results. It is also possible that those who responded were biased towards having noticed changes, whilst others who felt that all had remained the same were possibly less likely to engage in the questionnaire. Further research is also needed to quantify the impact of the COVID-19 pandemic on the delivery of routine immunizations worldwide to enable catch up programmes to be planned. However, the ability to assess this rapidly will vary by location based on the routine immunization data collection systems available.

Repeating this survey in the future would be informative and is already planned. This would primarily assess any changes in the key issues identified, particularly as the first wave of the pandemic evolves [1]. This repeat survey will aim to increase the response rate, particularly from countries now at the epicentre of the pandemic, and to explore further how to manage the challenges of immunization disruption. The epicentre has currently shifted to South America (Brazil) and, therefore, it would have been too early to capture the key issues, concerns and experiences of these countries with this initial survey [1]. Moreover, some areas in Sub-Saharan African are now seeing a significant increase in COVID-19 cases [43], which might lead to further changes in routine immunization services; on the other hand, other countries may have established effective mitigation strategies, after initially responding by downgrading services for fear of a rising epidemic, which, to date, has not materialised. Finally, it would be important to assess if lessons have been learned, further recommendations or guidelines have been published at the national or local level (or alterations to the existing ones) and the implementation of novel strategies has helped to overcome identified barriers.

## 5. Conclusions

As the COVID-19 pandemic continues, ongoing studies are needed to monitor routine immunization disruption and to further understand the reasons for disruptions to inform local vaccination catch-up programmes. Similarly, further work, beyond the modelling undertaken so far [10], is needed to understand the benefits of attending immunization appointments versus COVID-19 transmission risk. To address the current uncertainty reflected by this short study and experienced by healthcare providers, clearer and more tailored communication is urgently needed [8], particularly for LMICs. Improved efforts in communicating policies and conveying key messages should be targeted at pregnant women and parents, emphasizing the importance of attending routine vaccination services, despite the COVID-19 pandemic response. This is imperative for all settings, although our findings suggest there has been less official information provided for parents based in LMICs. Potential strategies in these settings may also need to focus on more community engagement to improve immunization uptake [48]. Accurate and up-to-date advice can be delivered using more user-friendly approaches, for example via social media, given the importance of accurate online messaging during the pandemic [49]. Effective communication strategies would serve to tackle misinformation, which has, unfortunately, been a worldwide concern during the pandemic [49,50]. Within our survey, “vaccine acceptance/conspiracy theories/anti-vaxx sentiments” were brought up more frequently by LMICs, specifically Sub-Saharan African countries (although not uniformly across settings). This may be consistent with the wave of vaccine hesitancy [47] and broader mistrust currently spreading across this region, with regards to both COVID-19 illness and potential COVID-19 vaccine trials to be undertaken in the future [51]. To date, the pandemic has largely been interpreted as an imported problem. This perception has in some places hampered acceptance of preventative measures [52] and might impact on future immunization campaigns, if a vaccine is tested and finally introduced.

Given the provider issues voiced by respondents in this questionnaire, increased efforts are needed to optimize logistics, both at a local and national level. This primarily includes supporting vaccine supply chains, reinforcing transport or infrastructure networks (for both vaccine delivery and user access/travel) and providing access to hand-washing facilities and PPE.

Prioritization of vaccination services is needed at local, regional and national levels, and recommendations have been provided by agencies such as the Joint Committee on Vaccination and Immunization in the United Kingdom and in Canada (both referenced by respondents in this survey) [32,33,53]. Midway during the pandemic, WHO also published a framework for governments to guide the conduct of vaccination campaigns [27]. This includes the importance of risk assessing the likelihood of outbreaks of vaccine-preventable diseases alongside the current transmission of COVID-19. General efforts explaining the safety measures implemented need to be communicated, in combination with the message that vaccination matters and saves lives, pandemic or not.

Beyond blanket recommendations, however, we may also need to focus on developing alternative, innovative and, in some cases, context-specific strategies to overcome or circumvent disruptions to service provision. Local measures should, therefore, ensure safety and practicality, but still be sufficiently flexible to rapidly adapt to the evolving situation, thereby avoiding suspension of key immunization programmes or services. Simple solutions such as aligning vaccination with other maternal and child health reviews, as well as robust infection control procedures, can enable the safe provision of key vaccines during the pandemic [37,54]. Alternative vaccination delivery mechanisms such as utilising pharmacies may also need to be considered [55]. More innovative examples include the development of drive-by or mobile clinics [56]; reassigning existing locations to facilitate vaccine delivery e.g., maternal antenatal clinics moved to the local football stadium, as described by one respondent, thereby overcoming issues with overcrowding and enabling social distancing; and a trial of “trace and immunise” systems (particularly using a digital interface) [39].

**Figure 3:**
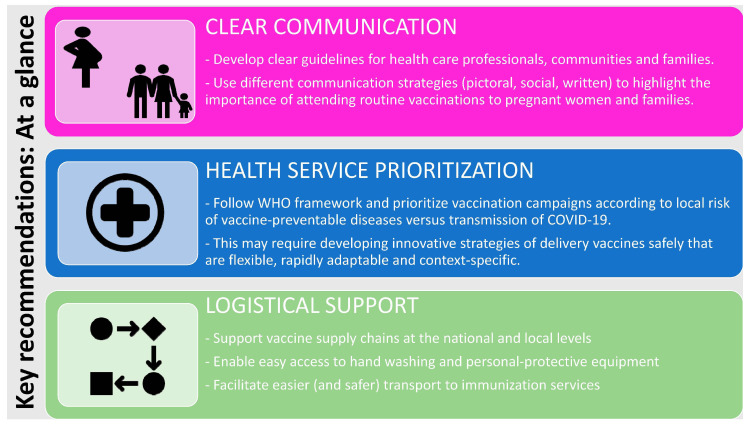
Key recommendations: At a glance. WHO: World Health Organization.

Based on our findings, the WHO framework and also the outcomes of the recent GAVI summit [57], we developed an “at-a-glance” visual summary of important recommendations for key stakeholders (Figure 3).

## Figures and Tables

**Table 1:** Respondent occupation.

Occupation	*N* (%)
Healthcare Professional (Doctor, nurse, midwife and private healthcare provider)	27 (56.3)
Laboratory based scientist	11 (22.9)
Public Health Official	4 (8.3)
Other *	6 (12.5)

* Other included: social scientists, pharmacists and students (in healthcare or clinical/social science research).

**Table 2:** Number of respondents who reported issues in delivering maternal, newborn and infant/childhood vaccines.

Country	Total Respondents (*n*)	Number of Respondents Reporting Issues Delivering Maternal Vaccines (%)	Number of Respondents Reporting Issues Delivery Newborn Vaccines (%)	Number of Respondents Reporting Issues Delivering Infant and Childhood Vaccines (%)
**All**	48	24 (50)	16 (33)	26 (54)
**LMIC**	36	19 (53)	15 (42)	19 (53)
**HIC**	12	5 (42)	1 (8)	7 (58)

LMIC, Low-and-middle-income country; HIC, high income country.

## Supplementary Materials

The following are available online at https://www.mdpi.com/2076-393X/8/3/556/s1, Table S1: Region, country of origin and occupation of respondents, and Questionnaire to IMPRINT members.
